# Synthetic pheromones and plant volatiles alter the expression of chemosensory genes in *Spodoptera exigua*

**DOI:** 10.1038/srep17320

**Published:** 2015-11-27

**Authors:** Xinlong Wan, Kai Qian, Yongjun Du

**Affiliations:** 1 Institute of Health and Environmental Ecology, Wenzhou Medical University, Wenzhou, Zhejiang 325035, China

## Abstract

Pheromone and plant odorants are important for insect mating, foraging food sources and oviposition. To understand the molecular mechanisms regulating pheromone and odorant signaling, we employed qRT-PCR to study the circadian rhythms of ABP, OBP, PBP, and OR gene expression in the beet armyworm, *Spodoptera exigua* and their responses after a pre-exposure to sex pheromone compounds or plant volatiles. The neuronal responses of male *S. exigua* to 20 chemical compounds were recorded at three specific time periods using the electroantennogram. The results showed a circadian rhythm in the expression profiles of some chemosensory genes in the antennae similar to their behavioral rhythm. The expression profiles of OR3, OR6, OR11, OR13, OR16, OR18, Orco, ABP2, OBP1, OBP7, and PBP1, and EAG responses to chemical compounds, as well as their circadian rhythm were significantly affected after exposure to synthetic sex pheromones and plant volatiles. These findings provide the first evidence that the gene expression of chemosensory genes and olfactory sensitivity to sex pheromones are affected by pre-exposing insects to pheromone compounds and plant volatiles. It helps to understand the molecular mechanisms underlying pheromone activity, and the application of sex pheromones and plant volatiles in mating disruption or mass trapping.

In natural conditions, the air is filled with a diverse of odors including pheromones as well as complex host plant odorants. Pheromones play a key role in the transmission of social information between insects. In general, pheromone is released by female adult, particularly in Lepidoptera, but there are also male-released sex pheromones^1^,^2^. Plants also emit large amounts of diverse volatile compounds into the air, which are variable by the species, but even the same plant may emit different compounds according to the physiological state^3^ or circadian rhythm^4^,^5^. For phytophagous insects, host plant volatiles may provide cues of food sources, habitats, and oviposition sites^6^,^7^, even in the third trophic level of insects, in which insects feed on other herbivores^8^. Because high numbers of plant volatiles are released into their natural habitats, moths have developed a complex olfactory system, which continues to evolve. Further, sex pheromones and plant volatiles exposure may cause changes in insect behaviors such as mating, calling, foraging, oviposition, and pheromone release, showing artificially mimicked response^9^,^10^. An insect’s olfactory system perceives specific signals in various ways based on the physiological status^11^,^12^,^13^,^14^,^15^. Thus, analyzing the effects of pheromones and plant volatiles will help unravel the operation mechanisms of olfactory systems^16^,^17^,^18^.

In insects, such as moths, pheromone information is transmitted by specialized olfactory receptor neurons (ORNs) to the macroglomerular complex (MGC), a male-specific part of the primary olfactory processing centre, the antennal lobe (AL). Plant odor information is transferred by general ORNs to sexually isomorphic ordinary glomeruli (OG)^19^. Smell identification in moths involves different gene families, including the odorant binding proteins (OBPs) and odorant receptors^20^,^21^. Hydrophobic odorant molecules in the air enter the hydrophilic lymph through micropores on the cuticle of the antennal olfactory sensors and bind OBP. The odorant molecule is then dissolved and transported to the olfactory neuron dendrites where it binds the odorant receptors^20^. During this process, ORs identify odor molecules and play an important role in transmitting signals, while the pheromone binding proteins(PBPs) and antennae binding proteins (ABPs) are commonly presumed to transport the odor molecules^22^. PBPs bind to sex pheromones and transfer them to receptors through the lymph of sensillum. Studies have shown that pheromones and plant volatiles may influence insect behavior by regulating gene expression of the olfactory system^23^,^24^,^25^.

The beet armyworm, *Spodoptera exigua* (Lepidoptera: Noctuidae), is one of the important agricultural pests worldwide, feeding on a wide range of plants including onion, beans, table beets, celery, cole crops, lettuce, potato, tomato, cotton, cereals, oilseeds, tobacco, flowers, and a multitude of weed species. It was found that in *S. exigua* OR6 was male-specific in the antenna, and that OR13 and OR16 were broadly activated by multiple pheromone components, but OR6 showed no response to any pheromone compound^26^. The expression profiles of OBPs (OBP1 – OBP11) in *S. exigua* were high in the antennae with OBP1-4 and OBP10 restricted to the female antennae, while the expression of OBP7 biased to the male-antennae^27^. Further, *in situ* hybridization indicated that OR3 in *S. exigua* had robust expression in the short trichoid sensilla, and narrowly tuned to detect *E*-*β*-farnesene and its derivatives^28^. Besides, PBP/GOBPs were shown to have primary expression in both male and female antennae but with different sex-biased expression patterns^29^. Together, these studies advance our understanding of the potential physiological functions of these proteins in *S. exigua*. These studies also provide a foundation of knowledge that may be adapted towards the development of non-polluting and low toxic insect aversion agents that may disrupt an insect’s olfactory system and reduce its ability to find the plant hosts and mate partners^30^.

We performed this study to understand the molecular mechanism underlying the interactions between sex pheromone and odorant signaling. Using quantitative real-time PCR (qRT-PCR) we determined the expression levels of selected ABP, OBP, PBP, and OR genes in *S. exigua* with or without pre-exposure to sex pheromone compounds and plant volatiles (green Chinese onion, *Allium fistulosum*). Further we also analyzed the electroantennogram (EAG) responses of *S. exigua* antennae to 20 chemical compounds. The results revealed the circadian rhythms of chemosensory receptor genes in this insect pest.

## Results

### The rhythm of ORs, ABPs, OBPs, PBPs gene expression in *S. exigua*

**Figure 1:**
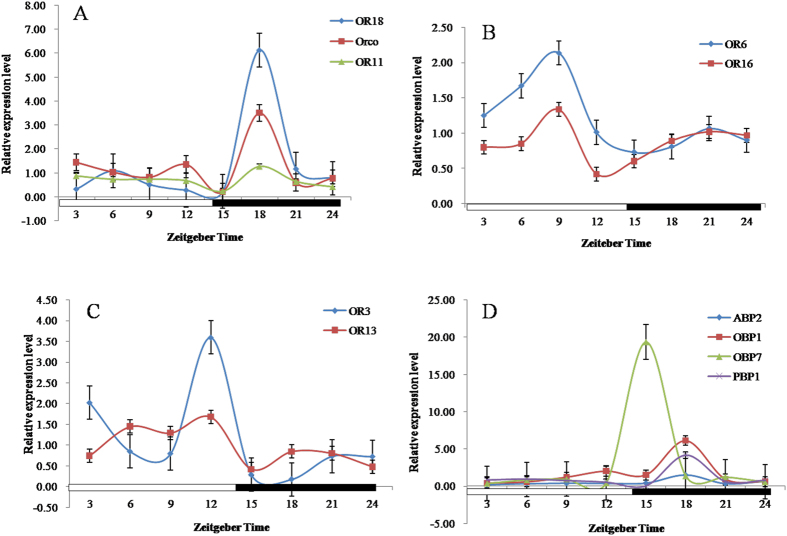
The circadian rhythms of ORs (**A–C**), ABP2, OBP1, OBP7, and PBP1 (**D**) gene expression in male *Spodoptera exigua*. All expression levels were calculated relative to those at ZT0. GAPDH gene was used to normalize the target gene expression and to correct for sample-to-sample variation.

The gene expression of ORs (OR3, OR6, OR11, OR13, OR16, OR18, and Orco) in *S. exigua* in the control group exposed to natural air was analyzed by qRT-PCR. The results showed that OR11, OR18 and Orco levels peaked at ZT18 (5.08, 25.50, and 15.91 folds higher than their lowest expression levels, respectively) (Fig. 1A); OR6 and OR16 expression reached maximum levels at ZT9 (2.92 and 3.12 folds higher than their lowest expression levels, respectively) (Fig. 1B); and OR3 and OR13 were maximum at ZT12 (12.82 and 4.00 folds higher than their lowest expression levels, respectively) (Fig. 1C). Thus, the peak of all OR gene expressions were at three different time points withOR11, OR18, and Orco reaching their peaks when the lights had been off for about four hours (ZT18). The expression levels of ABP2, OBP1 and PBP1 genes rose to the highest level at ZT18 (7.55, 14.93, and 208.5 folds higher than their lowest expression levels, respectively) (Fig. 1B). The maximum level of gene expression was achieved by OBP7 at ZT15 (50.92 folds higher than their lowest expression levels) (Fig. 1D). These results show that the peak gene expression of most tested genes in *S. exigua* occurs at ZT18, which is 1 -4 hours after initiation of darkness in the incubator indicating that these genes exhibit a circadian pattern.

### Effects of sex pheromones and plant volatiles on OBP, PBP, and ABP expression in *S. exigua*

**Figure 2:**
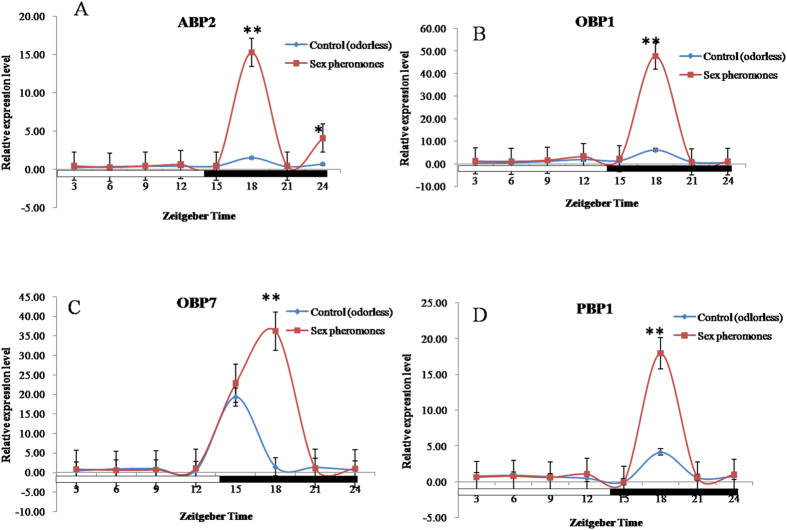
Effects of sex pheromone on the expression profile of ABP2 (**A**), OBP1 (**B**), OBP7 (**C**), and PBP1 (**D**) in male *Spodoptera exigua*. All expression levels were calculated relative to those at ZT0 as a control. GAPDH gene was used to normalize the target gene expression and to correct for sample-to-sample variation.

Analysis of gene expression by qRT-PCR showed that after pre-exposure to sex pheromones, the significant changes of expression of ABP2, OBP1, OBP7 and PBP1 genes occurred only at ZT18 (Fig. 2). Compared to the control group, in *S. exigua* exposed to the sex pheromones the expression levels of ABP2, OBP1, OBP7 and PBP1 genes at ZT18 were respectively 10.12-, 7.82-, 25.83-, and 4.31-fold higher, with significance (Fig. 2A–D). The expression level of ABP2 at ZT24 was also significantly higher (5.97 fold) than in the controls exposed to natural air at the same time point (Fig. 2A). Except for these two Zeitgeber time points (ZT18 and ZT24) the gene expression levels were not significantly different at other time points (Fig. 2A–D). The expression of most genes was relatively low but the expression levels of OBP7 in both control and pre-exposed *S. exigua* at ZT15 were markedly higher than the others although without a significant difference in fold-change between them (Fig. 2C).

**Figure 3:**
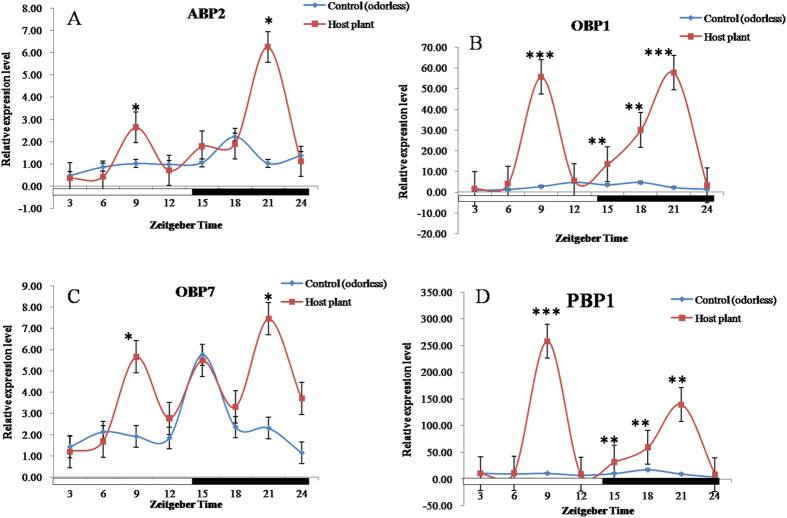
Effects of plant volatiles on the gene expression of ABP2 (**A**), OBP1 (**B**), OBP7 (**C**), and PBP1 (**D**) in male *Spodoptera exigua*. All expression levels were calculated relative to those at ZT0 as a control. GAPDH gene was used to normalize the target gene expression and to correct for sample-to-sample variation. Error bars signify significance of difference between control and treatments indicated by *or ^#^P < 0.05, **or ^##^P < 0.01, and ***P < 0.001.

On the other hand, compared to the control, *S. exigua* moths exposed to plant volatiles revealed the maximum expression of ABP2 at ZT21, which was 6.07-fold higher than that exposed to odorless air with significance (Fig. 3A); OBP1 at ZT9, ZT15, ZT18, and ZT21 significantly increased by 20.55, 3.82, 6.41, and 26.61 folds, respectively (Fig. 3B); OBP7 at ZT9 and ZT21 were significantly increased by 2.96 and 3.24 times, respectively (Fig. 3C); PBP1 at ZT9, ZT15, ZT18, and ZT21 were significantly increased by 24.33, 3.04, 3.45, and 14.90 folds, respectively (Fig. 3D).

### Effects of sex pheromones and plant volatiles on the olfactory receptor gene expression in *S. exigua*

**Figure 4:**
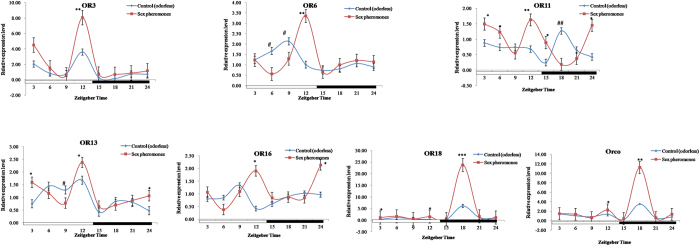
Effects of sex pheromones on the expression profile of OR genes in male *Spodoptera exigua*. All expression levels were calculated relative to those at ZT0 as a control. GAPDH gene was used to normalize the target gene expression and to correct for sample-to-sample variation. Error bars signify significance of difference between control and treatments indicated by *or ^#^P < 0.05, **or ^##^P < 0.01, and ***P < 0.001.

qRT-PCR analysis of olfactory receptor genes in *S. exigua* exposed to sex pheromones indicated that sex pheromones significantly induced the expression levels of OR3 and OR6 at ZT12 (increased 2.30 and 3.31 folds, respectively), OR11 at ZT3, ZT6, ZT12, ZT15, and ZT24 (advanced 1.86, 1.66, 2.40, 3.56 and 3.37 folds, respectively), OR13 at ZT3 and ZT12, and ZT24 (increased 2.13, 1.42 and 2.23 folds, respectively), OR16 at ZT12 and ZT24 (increased 4.55 and 2.21 folds, respectively), OR18 at ZT3, ZT12, and ZT18 (increased 3.55, 5.32, and 3.87 folds, respectively), and Orco at ZT12 and ZT18 (increased 1.65 and 3.20 times, respectively) (Fig. 4). The sex pheromones positively influenced the gene expression of all olfactory receptor genes tested, but with some exceptions. For instance, the expression levels of OR6 at ZT6 and ZT9, OR11 at ZT18, and OR13 at ZT9 in *S. exigua* exposed to sex pheromone were significantly lower than in the controls exposed to natural air at the same time points (Fig. 4).

**Figure 5:**
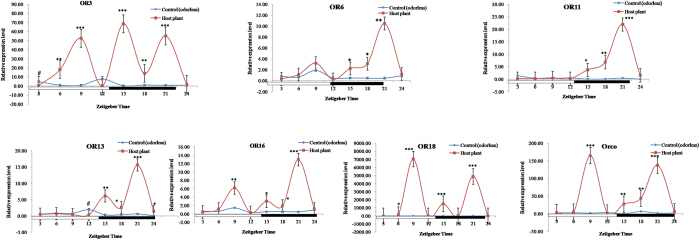
Effects of plant volatiles on the expression profile of OR genes in male *Spodoptera exigua*. All expression levels were calculated relative to those at ZT0 as a control. GAPDH gene was used to normalize the target gene expression and to correct for sample-to-sample variation. Error bars signify significance of difference between control and treatments indicated by *or ^#^P < 0.05, **or ^##^P < 0.01, and ***P < 0.001.

When compared to the controls, *S. exigua* exposed to host plant volatiles showed higher levels of olfactory receptor expression (Fig. 5). OR3 expression was significantly higher at ZT6, ZT9, ZT15, ZT18, and ZT21 (21.90, 78.37, 342.75, 15.75, and 76.69 folds, respectively), OR6 at ZT15, ZT18, and ZT21 (4.31, 6.00, and 21.41 folds, respectively), OR11 at ZT15, ZT18 and ZT21 (41.33, 61.82, and 46.57 folds, respectively), OR13 at ZT15, ZT18, ZT21, and ZT24 (19.81, 7.49, 26.12, and 36.00 folds, respectively), OR16 at ZT9, ZT15, ZT18, and ZT21 (1.87, 5.70, 3.25, and 25.92, respectively), OR18 at ZT6, ZT9, ZT15, and ZT21 (146.93, 13277.26, 5777.50, and 5527.77 folds, respectively), and Orco at ZT9, ZT15, ZT18, and ZT21 (90.05, 52.04, 6.55, and 75.58 folds, respectively). In contrast, the expression of OR3 at ZT3 and OR13 at ZT12 in control *S. exigua* unexposed to plant volatiles was significantly higher than those exposed to plant volatiles (Fig. 5).

### Effects of sex pheromones and host plant volatiles on EAG responses of *S. exigua* to chemical compounds

**Figure 6:**
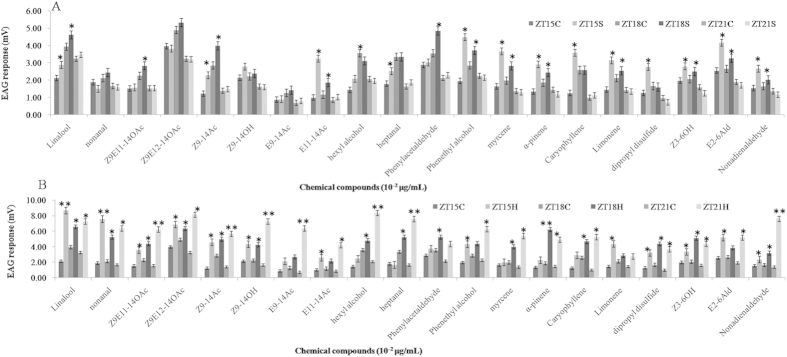
EAG responses of *Spodoptera exigua* moth to 20 different chemicals in the presence of sex pheromone compounds (**A**) and plant volatiles (**B**) at ZT15, ZT18, and ZT21. (**C**) control; S: sex pheromone compounds; H: plant volatiles. Error bars signify significance of difference between control and treatments indicated by *or ^#^P < 0.05, **or ^##^P < 0.01, and ***P < 0.001.

Considering that the expression levels of the tested chemosensory genes, in most cases, peaked at ZT15, ZT18, and ZT21, the EAG responses of *S. exigua* exposed to sex pheromones and host plant volatiles to chemical compounds were done only at the three ZT points. The EAG recordings indicated that sex pheromones and plant volatiles had different effects on EAG responses of *S. exigua* moths to different chemical compounds (Fig. 6). Overall, the sex pheromones and plant volatiles significantly elicited EAG responses of *S. exigua* to chemical stimuli at tested ZT points. However, the specific effects of same or different chemicals on the EAG responses of *S. exigua* were different at different ZT points. For example, at ZT18 the EAG response of *S. exigua* antenna to phenylacetaldehyde, Z9E11-14OAc and Z9E12-14OAc in the presence of sex pheromones was significantly higher than in odorless air although this difference was not observed at ZT15 and ZT21. Similarly, significantly high EAG responses were observed only at ZT15 and ZT18 when *S. exigua* were exposed to phenethyl alcohol, myrcene, α-pinene, linalool, Z9-14Ac, E11-14Ac, limonene, Z3-6OH, E2-6Ald, and nonadienaldehyde but not at ZT21. In addition, EAG responses to caryophyllene, hexyl alcohol, dipropyl disulfide and heptanal was significantly higher at ZT15, but not at ZT18 and ZT21. In contrast, the EAG responses of *S. exigua* antenna in the presence of sex pheromones to E9-14Ac, Z9-14OH and nonanal were not significantly different at any tested ZT point when compared to the control group exposed to odorless air (Fig. 6A). Together, these results indicate that the sex pheromone significantly increased the EAG responses of *S. exigua* moths to most chemical stimuli at ZT15 and ZT18, but not at ZT21, suggesting that the effect of sex pheromones on EAG responses of *S. exigua* is short-lived and may last only for up to 7 hours after dusk or dark.

In parallel, the EAG responses of *S. exigua* pre-stimulated by host plant volatiles were significantly higher than the control at the tested ZT points. For example, the EAG responses of *S. exigua* in the presence of host plant volatiles to linalool, nonanal, Z9E11-14OAc, Z9E12-14OAc, Z9-14Ac, Z9-14OH, dipropyl disulfide, Z3-6OH, and nonadienaldehyde were significantly higher than the control at all three tested ZT points. However, the remaining chemicals showed significant increase only at ZT21 (Fig. 6B). In contrast to the sex pheromones, the host plant volatiles significantly enhanced the EAG responses of *S. exigua* to all chemicals (Fig. 6B). These results indicate that the host plant volatiles may activate the neuronal behavior of *S. exigua*.

## Discussion

It is known that olfactory receptors expressed in the cell membranes of olfactory neurons are responsible for the detection of odor molecules. Activated olfactory receptors initiate a signal transduction cascade, which eventually produces a nerve impulse that is transmitted to the brain as smell. Many proteins associated with the olfactory system (OBPs, PBPs, ABPs, etc.) have antennae-specific or sex-specific expression patterns. Previous studies^26^ reported that some pheromone receptors in *S. exigua* were antennae-specific and that some in addition were sex-specific^31^. For example, OR6, OR13, and OR16 were found to be male-specific while OR11 was female-specific, however, OR3 was expressed in the antennae of both sexes. For this study, we chose the genes previously well characterized in the above reports. In this study, we investigated the gene expression rhythm of selected olfaction related genes in *S. exigua* and the EAG responses of the moths exposed to 20 different chemicals in the presence of sex pheromones or host plant volatiles. Our results showed a circadian pattern in the expression profiles of some olfactory genes and the influence of sex-pheromone and host plant volatiles in the EAG responses of *S. exigua* male antennae to different chemicals.

### Gene expression rhythm in *S. exigua* male moths

Circadian rhythms in physiological activities and insect behavior based on day/night alterations have been studied extensively^20^,^32^. Insects regulate their own rhythm by adjusting clock gene products, and this rhythm has also been reported in the insect olfactory organs^33^. For example, the EAG of fruit flies and cockroaches to host and other related odors showed a strong rhythm^34^. The peak mating behavior of the cockroach, *Leucophaea maderae,* occurred close to the light to dark transition time^35^. However, emergence of *Apis mellifera* workers from pupae showed no circadian based pattern but revealed strong rhythms later in life with volatile pheromones, the colony microenvironment, or both influencing the ontogeny of circadian rhythms in honey bees^36^. The peak in *Plodia interpunctella* calling was observed at dusk, whereas *Ephestia kuehniella* (both Lepidoptera: Pyralidae) calls specifically occurred at dawn, and the rhythm of male locomotor activity corresponded well with the sexual activity of females, reaching the peak at dusk and at dawn in *P. interpunctella* and *E. kuehniella*, respectively^37^. These studies indicate that the biological clock plays an important role in the regulation of insect olfactory behavior.

In our study, the expression levels of all tested genes related to sex pheromones and odorants in *S. exigua* exposed to odorless air (control) revealed that the majority of them (6 among 11 genes) reached the peak at ZT18, while others maximized at ZT15 (1 gene), ZT12 (2 genes), and ZT9 (2 genes) (Fig. 1). ZT18 is the time when the lights had been off for about four hours suggesting that the physiological states of *S. exigua* may become most active approximately four hours after the light/dark transition. Since physiological states are reflected by behaviors such as locomotion, copulation, oviposition, eclosion, and pupation our results indicate that the biological behaviors of *S. exigua* that are regulated by ORs, OBPs, ABPs, and PBPs such as copulation and oviposition could likely occur about four hours after dark. This is consistent with previous studies in *S. exigua* and other moths that mate mostly in the dark to avoid natural enemies. Peak mating in *Holcocerus hippophaecolus* and *Holcocerus vicarious* occurred at 21:00 and 23:00 hours, respectively^38^,^39^ while copulation of *Syllepte derogata* occurred after lights were off and the peak appeared to be at 20:00–22:00 hours^40^. Most *S. exigua* have been reported to mate at 02:00–07:00 hours^41^. However, some insects such as *Hyphantri acunea* displayed a bimodal mating pattern between 03:00–04:00 and 04:00–05:00 hours^42^. Oviposition in insect moths also mostly occurs in scotophase of next day after copulation. For example, ovipositionin *S. derogate* Fabricius, *H. hippophaecolus* and *S. exigua* occurred at 20:00–22:00, 20:30–22:00 and 00:00–05:00 hours, respectively the next day after copulation^38^,^40^,^41^.

### Effects of the chemosensory gene expressions on olfactory system and behavior in *S. exigua*

Innate behavior in animals can be influenced by biotic and abiotic factors, such as the environment, experience, or physiological status^43^. Pheromones and plant volatiles in the environment can alter neural response threshold by causing changes in the behavior of an organism^18^. A recent report demonstrated an intense interaction between the pheromone and odorant subsystems in AL moths^44^. Moreover, in the field the plant-derived kairomones, pear ester could cause a low level of synthetic lure trap disruption and *E*-β-farnesene could disrupt mating^45^. However, the molecular mechanism behind the cause of these behavioral changes is poorly understood. Our results indicated that the expression levels of plant odorant and sex pheromone transport-related proteins (PBP, ABP, OBP, and OR) in *S. exigua* moths exposed to host plants and sex pheromone compounds were significantly higher than the controls at some time points. Intriguingly in general, the sex pheromones showed some relatively higher effects on expression levels of OBP/PBP/ABP (increased 10.81 folds in average) than those of ORs (increased 2.86 folds in average), while plant volatiles were the exact opposite of the sex pheromones (ORs: increased 991.93 folds in average; OBP/PBP/ABP: increased 10.93 folds in average), even though there were few exceptions. This may be related to different functions of the two kinds of gene families in insects. Further studies to confirm this might be necessary. The circadian rhythms of these gene expression patterns were obviously modulated by the plant odorants and sex pheromones suggesting that plant odorants and sex pheromones coeffect the level of gene expression in *S. exigua* moths (Figs 2, 3, 4, 5). This result is consistent with the EAG responses showing the positive effects of sex pheromones and host plant odorants on the olfactory behavior of *S. exigua* to most chemicals (Fig. 6). In detail, the significant effects of sex pheromones on OBP/PBP/ABP and ORs mainly occurred at ZT18 and Z12, (Figs 2 and 4), while the significantly increased EAG responses of *S. litura* antennae in presence of sex pheromone to 20 chemicals also mainly occurred at ZT18 (Fig. 6a). Likewise, the significant effects of plant volatiles on OBP/PBP/ABP and ORs mainly occurred at ZT15 to ZT21 (Figs 3 and 5), while the significantly increased EAG responses of *S. litura* antennae in presence of plant volatiles to 20 chemicals also almost occurred at the three ZT points (Fig. 6b). Wind tunnel experiments verified that pre-stimulation through sex pheromone compounds may enhance sensitivity of the male moth sex pheromone response with the male moth flying or crawling more easily to the odor source even in the day^46^,^47^. Electrophysiological recordings showed increased response to sex pheromones in the antennal lobe neurons^47^. Another study also showed that pre-exposure to the sex pheromone, codlemone, increased the behavioral response of codling moth males to pear ester using wind tunnel, supporting the idea that male attraction to pear ester and codlemone is mediated through the same sensory channels^48^. It is believed that such pre-stimulation impacts the brain, but further electrophysiological recordings showed that pre-stimulation of the male moth improves the peripheral nervous system response to sex pheromones^49^.

Host plant volatiles have also shown to synergize the behavioral responses of insects to their sex pheromones and host-related odorants^18^. For example, EAG responses of oblique-banded leafrollers, *Choristoneura rosaceana*, and red-banded leafrollers, *Argyrotaenia velutinana,* to the major component of their sex-attractant pheromone (Z11-14:Ac) and plant odorants in the presence of nine of their host-related plant volatiles were increased thus indicating the octopamine-mediated sensitization^50^. Wind tunnel experiments also demonstrated that apple volatiles synergize attraction of the male codling moth, *Cydia pomonella*, to the sex pheromone (E, E)-8,10-dodecadien-1-ol^51^. Deng *et al.*^52^ found that exposure to volatile compounds stimulated strong EAG responses in male *S. exigua* to sex pheromones and exposure to volatile compounds produced by host plants also enhanced the orientation response of *S. exigua* males to sex pheromone sources. Nevertheless, recent studies suggested a negative effect of plant odorants on the responses of moths to sex pheromones. Party *et al.*^12^ analyzed pheromone olfactory receptor neuron (Ph-ORN) responses of *Spodoptera littoralis* in the presence of 4 monoterpenes to the major pheromone component, Z9E11-14:Ac. Their results showed that linalool reversibly reduced the firing response to Z9E11-14:Ac and produced an off effect, while geraniol, geranyl and linalyl acetates reduced the responses to Z9E11-14:Ac with a longer time course confirming that plant compounds may modulate the intensity and temporal coding by Ph-ORNs. This modulation could positively affect mate location at high pheromone density especially near a pheromone source. A volatile plant background on the walking response of male *S. littoralis* to the female pheromone was found to distract the orientation toward the sex pheromone and the effect on locomotion correlated with the capacity of the plant compound to antagonize pheromone detection by olfactory receptor neurons suggesting that background odors may mask pheromone signal^13^.

In conclusion, we report here the impact of sex pheromone and plant odorants on the gene expression of *S. exigua*. Thus, this is the first report to show the circadian rhythm like expression of chemosensory genes in *S. exigua* and effects of sex pheromones and plant volatiles on the expression levels of olfactory proteins, which therefore modify the olfactory profile of *S. exigua* to odorants in the environment. This data will help advance understanding of the physiological functions of chemosensory proteins in *S. exigua* and the molecular mechanism on both of mating disruption by pheromone and effect of plant background on the olfactory recognition of insect to sex pheromone, and contribute to the design of new strategies to control the beet armyworm.

## Methods

### Insects

Male *S. exigua* pupae were purchased from Keyun Biological Co. Ltd. in Henan Province, China, and maintained in an artificial intelligent climate chamber at 28 °C, 65% humidity and 14:10light:darkperiod. After emergence, the adults were fed 10% sucrose.

### Experimental protocol

Adult male *S. exigua* were raised in four different odorless insect incubators under identical conditions (light:dark 14:10; photophase: Zeitgeber Time (ZT) 0–ZT14; scotophase: ZT14–ZT24; Temperature: 30 °C; Humidity: 65%).The four incubators were randomly divided into two groups with 2 in each group. One incubator in group 1 received the sex pheromone treatment with synthetic *S. exigua* sex pheromone lure (Ningbo Newcon Inc.) and one incubator in group 2 received the host-plant treatment with green Chinese onion. The remaining 2 incubators were used as the respective controls. The four incubators were kept far enough from each other so that the exchange of gas molecules between them can be ignored. The pheromone or host plant volatiles were placed in the incubators at ZT0, and the pheromone or volatile was not removed in the incubator until the work ended.

Antennae from *S. exigua* males in all four incubators were collected at ZT3, ZT6, ZT9, ZT12, ZT15, ZT18, ZT21, and ZT24. Three replicates were collected for each time point and 25 pairs of antennae were collected for each replicate. Antennae from each group were immediately homogenized in Trizol on ice, and stored at −80 °C for RNA extraction. Antennae collection and RNA extraction during the scotophase was executed in the dark under red light.

### RNA extraction, cDNA synthesis and quantitative real-time PCR

Total RNA was extracted using RNAiso Plus (Takara Biotechnology Co., Ltd.) and DNase was added for elimination of the potential DNA in RNA according to the manufacturer’s protocol. The concentrations of extracted RNA were determined using Nanodrop 1000 (Thermo Fisher). cDNA was synthesized using PrimeScript RT-PCR in 20 μL reaction volumes (Takara) containing 1 μg total RNA, 1 μL Prime-Script™ Enzyme Mix, 1 μL OligodT primer (50 μmol/L), 4 μL 5 × PrimeScript™ buffer and RNase free water. The reaction conditions were: 37 °C for 25 min and 85 °C for 5 s. All samples were replicated 3 times.

To study the effects of the sex pheromone on gene expression levels and circadian rhythm of *S. exigua*, we selected the following genes with high expression level in *S. exigua*: some ORs (OR3, OR6, OR11, OR13, OR16, OR18, and Orco), OBPs (OBP1 and OBP7), 1 PBP (PBP1), and 1 ABP (ABP2).The house keeping gene, GAPDH, was used as the reference gene based on previous qRT-PCR studies^26^,^28^,^31^,^53^ and our preliminary work showing that the expressions of GAPDH were stable across all time points (data not shown). The primer sequences used in qRT-PCR are shown in Table 1. The amplicon sizes ranged from 100 bp to 200 bp depending on different genes and were verified on gel. One amplicon of all tested genes was sequenced for further confirmation. The antennal cDNA extracted at various time points after exposure of *S. exigua* to the sex pheromone, plant volatile and natural air (control) were used as templates for the qRT-PCR reactions. All reaction volumes were 20 μL and included 10 μL SYBR Premix ExTaq mixture (Takara Biotechnology Co., Ltd.), 1 μL each of the forward and reverse primers, 1 μL cDNA and 7 μL RNase free water. Three replicates were set for each sample. The amplification was performed in a Bio-Rad qRT-PCR instrument as follows: initial denaturation at 94 °C; 45 cycles at 94 °C for 15 s, 58 °C for 40 s, and 72 °C for 20 s. Relative gene expression was calculated using the 2^− ΔΔCt^ method.

### Electroantennogram (EAG) recordings

Recordings of electrical activity of whole-antennae in response to volatile stimuli were made according to standard techniques. A male moth was stabilized in a 1-mL plastic pipette with a cut tip to allow only the antennae to protrude through the opening. The tip of one of the antenna was cut, and a recording electrode filled with Beadle-Ephrussi Ringer was placed in contact with the cut surface and base of the antenna. An Ag/AgCl wire serving as a ground electrode was inserted into the insect’s abdomen. The antenna was continuously flushed with moistened air stream, which was purified by a charcoal filter in a glass tube (8 mm i.d.). The outlet of the tube was about 20 mm from the antenna. The stimulus was injected into the air stream through a Pasteur glass tube 15 cm upstream from the antenna. The stimulation was delivered at a flow rate of 5 mL/sec in 0.5-sec puffs using a stimulation device (Syntech, The Netherlands). The signal was amplified using a high impedance amplifier, as well as stored and analyzed with the EAG2000 software. Antennae of controls and *S. exigua* exposed to sex pheromones and host plant volatiles for ZT15, ZT18, and ZT21 were challenged with 20 volatile chemicals (Table 2) selected from flowers, host or non-host plants, and sex pheromones from other moths. Each chemical was dissolved in paraffin oil and tested at 10^−2^ μg/mL. A 10 μl aliquot of paraffin oil on the filter paper was used as the control. The responses of antennae from ten male moths were individually tested for each treatment. Recordings during the scotophase were performed under red light.

### Data Analysis

The multiple comparisons were executed by One-Way ANOVA followed a least significant difference (LSD) test (significance level: P < 0.05) with SPSS10.0.1 software (SPSS Inc.), with P < 0.05 marked as *, P < 0.01 as ** and P < 0.001 as ***.

## Additional Information

**How to cite this article**: Wan, X. *et al.* Synthetic pheromones and plant volatiles alter the expression of chemosensory genes in *Spodoptera exigua. Sci. Rep.* **5**, 17320; doi: 10.1038/srep17320 (2015).

## Figures and Tables

**Table 1:** List of primers used in qRT-PCR.

Primer name	Primer sequence (5′ to 3′)
SexiOR3-F	ATCTGGACAACTGGCGTGA
SexiOR3-R	ATGGTCTATCAATGGCATCTCC
SexiOR6-F	CTACCATTACGATTGAAGGAGG
SexiOR6-R	TAATAGCAGACCAACATCGGAC
SexiOR11-F	AAGGCTCTTGGCGAGAAAACT
SexiOR11-R	AGTTCCAAAAAGCCGAATCTAT
SexiOR13-F	CTGGAGGGAATCGAGAATGT
SexiOR13-R	AAGTGTACCCGGTATCAAGGA
SexiOR16-F	CTACCATTACGATTGAAGGAGG
SexiOR16-R	TAATAGCAGACCAACATCGGAC
SexiOR18-F	AGAGACCACCAAGATGCGTTAGAC
SexiOR18-R	GCCGACCACCCCGAATGAC
SexiOrco-F	ATGGAACCAGTCAAACAGTCACCCTC
SexiOrco-R	ACCCAGCAAACAACAGACAGCACA
SexiABP2-F	CGTTGACAGCACTATGGCGTTC
SexiABP2-R	ACAGCAATTTGGCTCTATCACACC
SexiOBP1-F	TGGCATCAACGCTTGCTTCTTAC
SexiOBP1-R	GGCTTTATCGGCATCGTATTCTCC
SexiOBP7-F	GCAGGGGAGAGGAATGGAAAGC
SexiOBP7-R	CGACATCAGGCATCTAAGGAACTTG
SexiPBP1-F	TGGCAAAGAAGTTGGACC
SexiPBP1-R	TGCTCGCATTCGTGGAT
SexiGAPDH-F	GACAACCACTCATCTATCTTCG
SexiGAPDH-R	AACATTTATCTCTACAACGCAATC

**Table 2:** Compounds used in the EAG tests.

Chemicals	Molecular Weight	Purity	Source
Z9E11-14OAc	252.39	≥92.0%	Bedoukian Research
Z9E12-14OAc	252.39	≥93.0%	Bedoukian Research
Z9-14OH	212.38	≥96.0%	Bedoukian Research
Z9-14Ac	254.41	≥95.0%	Bedoukian Research
E9-14Ac	254.41	≥96.0%	Bedoukian Research
E11-14Ac	254.41	≥95.0%	Bedoukian Research
Z3-6OH	100.16	≥98.0%	Sigma Aldrich
E2-6Ald	98.15	≥95.0%	Sigma Aldrich
Caryophyllene	204.35	≥80.0%	Sigma Aldrich
Hexyl alcohol	102.17	≥98.0%	Sigma Aldrich
Dipropyl disulfide	150.31	≥98.0%	Sigma Aldrich
Heptanal	114.19	≥95.0%	Sigma Aldrich
Nonanal	142.24	≥95.0%	Sigma Aldrich
Linalool	154.25	≥98.0%	Sigma Aldrich
Phenethylalcohol	122.16	≥99.0%	Sigma Aldrich
Phenylacetaldehyde	120.15	≥95.0%	Sigma Aldrich
Myrcene	136.23	≥95.0%	Sigma Aldrich
α-pinene	136.23	≥99.0%	Sigma Aldrich
Limonene	136.23	≥99.0%	Sigma Aldrich
Nonadienaldehyde	138.2	≥97.0%	Bedoukian Research
